# Split-Cre Complementation Restores Combination Activity on Transgene Excision in Hair Roots of Transgenic Tobacco

**DOI:** 10.1371/journal.pone.0110290

**Published:** 2014-10-17

**Authors:** Mengling Wen, Yuan Gao, Lijun Wang, Lingyu Ran, Jiahui Li, Keming Luo

**Affiliations:** 1 Key Laboratory of Eco-environments of Three Gorges Reservoir Region, Ministry of Education, Institute of Resources Botany, School of Life Sciences, Southwest University, Chongqing, China; 2 Chongqing Key Laboratory of Transgenic Plant and Safety Control, Southwest University, Chongqing, China; 3 Key Laboratory of Adaptation and Evolution of Plateau Biota, Northwest Institute of Plateau Biology, Chinese Academy of Sciences, Xining, China; Clemson University, United States of America

## Abstract

The Cre/loxP system is increasingly exploited for genetic manipulation of DNA *in vitro* and *in vivo*. It was previously reported that inactive ‘‘split-Cre’’ fragments could restore Cre activity in transgenic mice when overlapping co-expression was controlled by two different promoters. In this study, we analyzed recombination activities of split-Cre proteins, and found that no recombinase activity was detected in the *in vitro* recombination reaction in which only the N-terminal domain (NCre) of split-Cre protein was expressed, whereas recombination activity was obtained when the C-terminal (CCre) or both NCre and CCre fragments were supplied. We have also determined the recombination efficiency of split-Cre proteins which were co-expressed in hair roots of transgenic tobacco. No Cre recombination event was observed in hair roots of transgenic tobacco when the NCre or CCre genes were expressed alone. In contrast, an efficient recombination event was found in transgenic hairy roots co-expressing both inactive split-Cre genes. Moreover, the restored recombination efficiency of split-Cre proteins fused with the nuclear localization sequence (NLS) was higher than that of intact Cre in transgenic lines. Thus, DNA recombination mediated by split-Cre proteins provides an alternative method for spatial and temporal regulation of gene expression in transgenic plants.

## Introduction

The phage P1 Cre recombinase is a member of the tyrosine recombinase family and catalyzes site-specific DNA recombination between tandem 34-bp loxP DNA sequences [1], [2]. If two loxP sites are introduced in the same orientation into a genomic locus, Cre-mediated recombination will result in the deletion of the loxP-flanked DNA sequences. The Cre/loxP recombination system is a sophisticated tool for general knockouts, conditional knockouts and reporter strains, and has been widely used in a variety of organisms, including yeasts [3], [4], plants [5]–[9] and animals [1], [9]–[12]. In general, Cre recombinase is expressed under the control of a cell-or tissue-specific promoter to achieve targeted gene knockout in a spatial-temporal fashion [13]–[15]. However, it is not always facile to find a gene-specific promoter to control expression of the Cre recombinase specifically in a desired cell type.

Active protein can be cleaved into two inactive fragments which can directly re-associate to restore activity [16]–[18]. Cre recombinase consists of 343 amino acids that form two distinct domains. The N-terminal domain encompasses residues 20–129 and contains five α-helical segments linked by a series of short loops. The C-terminal domain contains amino acids 132–341 and harbors the active site of the enzyme [19]. Based on its protein structure, the Cre recombinase has previously been split into two complementation polypeptides at different break points such as Asn59/Asn60, Leu104/Arg106 and Gly190/Gly191[16], [20], [21], [22], [23], and the recombination activity could be reconstituted *in vivo*. In a previous report, Cre recombinase was divided into two independent polypeptides, a-NH_2_ terminal with the amino acids 19–59 and b-COOH terminal with the amino acids 60–343 [24]. When two fragments with overlapping amino acid sequences of the Cre gene were co-expressed, recombinase activity was restored even without the addition of dimerization modules [24], [25]. Maruo et al. (2008) systematically analyzed the efficiency of Cre complementation by screening multiple dimerization modules in Cos7 cells and primary neurons [26]. To improve the efficiency of split-Cre a-complementation, two inactive fragments were reconstituted by the leucine zipper domain dimerization [20]. However, reassembling split-Cre protein has not yet been reported in higher plants. In this study, we used the a-complementation approach to split Cre and introduce the two inactive fragments into transgenic tobacco (*Nicotiana tabacum* cv. Xanthi). Our experiments revealed that no recombination activity was detected in transgenic tobacco hair roots when individual N- or C-terminal fragments of Cre recombinase gene were expressed. While Cre enzyme activity was able to be restored *in vivo* when co-expressed these polypeptides. Therefore, we provide a new strategy for DNA recombination and gene expression regulation in plants.

## Materials and Methods

### Plant material and bacterial strains

*Nicotiana tabacum* cv. Xanthi was grown on Murashige and Skoog medium in a greenhouse under an 18/6h(light/dark)photoperiod at 25°C.

*Escherichia coli* strain DH5a was used as the recipient for transformation, genetic manipulation and production of plasmid DNA for sequencing. *E. coli* strain BL-21 (DE3) was used for protein expression. The disarmed *Agrobacterium rhizogenes* strain C58C1 was used for tobacco transformation.

### Vector construction

The N- (amino acids 1–59) and C-terminal (aa 60–343) moieties of Cre recombinase [20], [21] and full-length Cre were amplified by PCR using primers NCre-F, NCre-R, CCre-F and CCre-R (listed in Table 1) with *Eco*RI and *Hin*dIII restriction sites at their 5′ ends. The NCre and CCre gene fragments were cloned into the multiple cloning sites of prokaryotic expression vector pMAL-C2X digested with the same enzymes, respectively. Cre gene fragment was cloned into prokaryotic expression vector pET-28a. All the recombinant plasmids were then transformed into host cells *E. coli* BL-21 (DE3).

**Table 1:** DNA oligo sequences utilizes in this report.

Primer name	Primer sequence (5'-3')	Restriction enzyme site
NCre-F	cggaattcatgtccaatttactgaccgtac	*Eco*R I
NCre-R	cccaagcttctaattcaacttgcaccatgcc	*Hin*d III
CCre-F	cggaattcatgaaccggaaatggtttcccg	*Eco*R I
CCre-R	cccaagcttctaatcgccatcttccagca	*Hin*d III
pX6-NCre-F	gaagatctatgtccaatttactgaccgtac	*Bgl* II
pX6-NCre-R	catgggatccctaattcaacttgcaccatgcc	*Bam*H I
pX6-NLS-NCre-F	gaagatctcccaagaagaagaggaaggtgatg	*Bgl* II
	tccaatttactgaccgtac	
pX6-CCre-F	atgaaccggaaatggtttcccg	/
pX6-CCre-R	ctaatcgccatcttccagca	/
pX6-NLS-CCre-F	atgcccaagaagaagaggaaggtgaaccggaa	/
	atggtttcccg	
pCa-F	gatgacgcacaatcccactatcc	/
pCa-R	gtacagactagttcgtcggttctg	/
F1	cgggatccgaacgtgcaaaacaggctct	/

To construct the plant binary vectors, we synthesized the loxP-nos-loxP fusion sequences by a commercial company (Huada, Shenzhen, China). Sequences were as follows:5′-CGGGATCCGC**ATAACTTCGTATAATGTATGCTATACGAAGTTAT**AGATCTTC*CGTTCAAACATTTGGCAATAAAGTTTCTTAAGATTGAATCCTGTTGCCGGTCTTGCGATGATTATCATATAATTTCTGTTGAATTACGTTAAGCATGTAATAATTAACATGTAATGCATGACGTTATTTATGAGATGGGTTTTTATGATTAGAGTCCCGCAATTATACATTTAATACGCGATAGAAAACAAAATATAGCGCGCAAACTAGGATAAATTATCGCGCGCGGTGTCATCTATGTTACTAGATCGGG* **ATAACTTCGTATAATGTATGCTATACGAAGTTAT**GGATCCCG-3′. The bold letters represent the loxP site (34 bp) and the italic letters represent the NOS terminator sequence (253 bp). The underlined letters show the restriction enzyme sites: *Bam*HI, *Bgl*II and *Bam*HI, respectively. The *Bam*HI-digested loxP-nos-loxP fragment was ligated to the binary vector pCAMBIA1305.1 which was digested with *Bgl*II, producing the vector ploxP. The NCre, CCre and full-length Cre fragments were amplified by PCR and inserted respectively into the vector pCXSN [27], respectively. And then the 35S-NCre-nos, 35S-CCre-nos, 35S-Cre-nos fusion gene segments were excised from the resulting pCXSN vectors with *Eco*RI/*Hin*dIII digestion and then ligated into the corresponding sites of the ploxP vector, producing the vectors pCre, pNCre and pCCre. To add an extra nuclear localization signal (NLS) sequence to N-terminus of Cre, NCre and CCre, the oligos (nNCre-F and nCCre-F) (Table 1) were utilized. PCR fragments were cloned into the ploxP vector to produce pnCre, pnNCre and pnCCre, respectively. To construct the vectors pCCre-nNCre and pnCCre-nNCre, the loxP-nNCre-loxP fusion gene fragment was amplified by PCR and ligated into the pCCre and pnCCre by digesting with *Bgl*II, respectively. All the plant binary vectors were introduced into the *A. rhizogenes* strain C58C1 *via* a simple freeze/thaw transformation method [28].

### Expression and purification of proteins

The *E. coli* strain BL-21 (DE3) was transformed with expression vectors containing NCre, CCre and full length Cre proteins. A colony of the transformed cells was cultured in LB medium with ampicillin (100 µg/mL) at 37°C with 180 rpm until OD_600_  =  0.6. Protein expression was induced by isopropyl β-D-thiogalactoside (IPTG) at 0.1 mM. Incubation was continued to culture at 25°C with 180 rpm for 4 h before the bacteria were harvested by centrifugation. The cells were resuspended in phosphate buffered saline (PBS) after washing. Clear lysate was obtained after centrifugation because NCre, CCre and full length Cre proteins are expressed in the soluble fraction. The purity and relative concentrations of these proteins were examined by 12% SDS-PAGE [29], [30]. All of the purified proteins were stored at -80°C after adding glycerol with a 1∶1 ratio.

### *In vitro* assays of recombination activity

In order to detect the recombination activity of purified proteins, including split-Cre (NCre and CCre) and full-length Cre, the plasmid ploxP-CCre629, in which a 1200-bp DNA fragment was flanked by two loxP recognition sites in the same orientation, was digested at 37°C for 1 h by the purified proteins. The reaction system was as follows: 1 µL 10 × Buffer L (TaKaRa, Dalian, China), 5 µL plasmid (90 ng/µL), 3 µL purified protein and 1 µL ddH_2_O, total 10 µL. As a control, the plasmid ploxP-CCre629 was also digested with *Hin*dIII and *Bam*HI at 37°C for 1 h. The digested product was used for DNA electrophoresis.

### Transformation of tobacco plants

*A. rhizogenes* strain C58C1 with the plant binary vectors was incubated in liquid YEP medium supplemented with 50 mg/L kanamycin and 40 mg/L rifampicin at 28°C and 180 rpm until the cultures reached an optimal density of approximately 0.6–0.8 at OD_600_ [31]. After centrifuged for 10 min at 4,000 rpm and 4°C,the cultures were resuspended with an equal volume of liquid MS medium (MS medium, 100 µmol/L acetosyringone; pH5.8) [32]. Tobacco transformation was performed using the leaf disc method as described previously [33]. After growing on the co-cultivation medium (MS medium, 100 µmol/L acetosyringone, 30 g/L sucrose, 6 g/L agar, pH5.8) in darkness at 25°C for 2 days, the leaf discs were transferred to a selective medium (MS medium, 10 mg/L hygromycin, 150 mg/L rifampicin, 30 g/L sucrose, 6 g/L agar, pH5.8) under a photoperiod of 16:8 (light:dark) h at 25°C.

### GUS staining assay

Activity of β-glucuronidase (GUS) in transgenic hair roots was determined by a GUS histochemical staining assay [34]. Transgenic hair roots were placed in 1 mM X-gluc (5-bromo-4-choloro-3-indolyl-b-glucuronic acid) solution and incubated at 37°C overnight and was subsequently recorded photographically.

### RNA extraction and reverse-transcriptase PCR (RT-PCR)

Total RNA of hair roots was extracted using TRIzol Reagent (Invitrogen, Beijing, China) according to the manufacturer's instructions. First-strand cDNA was synthesized from 1 µg of total RNA using PrimeScript RT reagent Kit with gDNA Eraser (TaKaRa, Dalian, China). RT-PCR was performed as previously described for genomic PCR using gene-specific primers (Table 1) for different genes. Reaction products were resolved by electrophoresis in 1.5% agarose gel. A pair of specific primers for *18S* of *N. tabacum* [35] were used in a control reaction.

### DNA extraction and molecular analysis of transgenic plants

Genomic DNA was extracted from transgenic and untransformed control hair roots using the modified CTAB extraction method as described previously [36]. Putative transgenic hair roots were screened preliminarily to confirm the presence of the transgenes by PCR method [37]. Two gene-specific primers pCa-F and pCa-R (Table 1), which flanked two loxP sites, were designed for detection of transgene excision. PCR was conducted at 94°C for 5 min, followed by 30 cycles of 94°C for 30 s, 56°C for 30 s, 72°C for 1 min, and a final extension step at 72°C for 10 min. The PCR products were loaded on 1% (w/v) agarose gel and visualized after ethidium bromide staining. The PCR fragment was cloned into pMD19 vector (TaKaRa, Dalian, China) and sequenced by Beijing Genomics Institute.

## Results

### *In vitro* assays for recombination activity of split-Cre complementation

**Figure 1:**
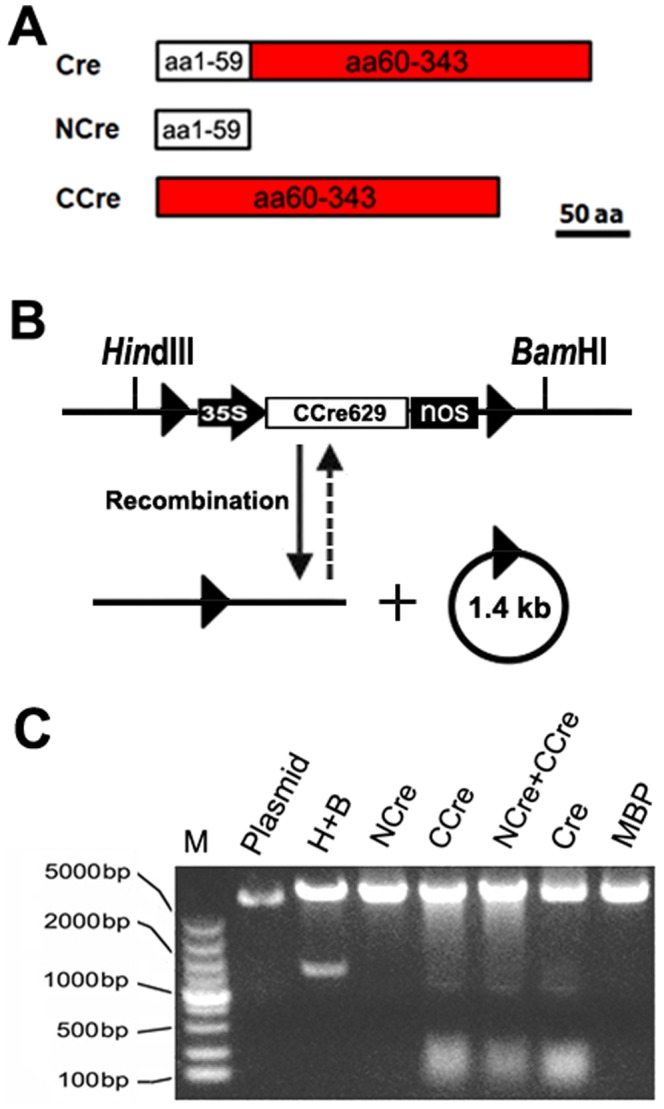
Digram of the split-Cre model and *in vitro* recombination of Split-Cre protein. **A: Digram of the split-Cre model.** The intact Cre was designed to be split at the 60th amino acid residue. Two molecules of split-Cre were named NCre and CCre respectively. **B: Structure of the substrate catalyzed by purified protein. C: Recombination assay of Split-Cre protein** ***in vitro*** **.** M: DL5000 Marker; Plasmid: 2µl plasmid (90 ng/µl) of pLoxp-Ic-CCre629. The plasmid was respectively digested by *Hin*dIII and *Bam*HI (H+B), split protein NCre (NCre), split protein CCre (CCre), combination of split protein NCre and CCre (NCre + CCre), intact protein Cre (Cre) and MBP. Plasmid and MBP were used as negative control, H+B digestions were used as positive control. MBP tag was used to purify fusion proteins.

To establish a split-Cre complementation system, the coding sequence of Cre recombinase was cleaved into two complementation-competent fragments, named NCre (amino acids residues 1-59) and CCre (amino acids residues 60-343) (Fig. 1A), according to previous reports [20], [21]. These split-Cre- and Cre-genes were cloned into the expression vector pMAL-C2X and pET-28a (Novagen) and recombinase proteins were produced in reticulocyte lysates. Under the induction of isopropyl β-D-thiogalactoside (IPTG), Split-Cre and full-length Cre proteins were purified to detect the recombination activity (Fig. S1). *In vitro* excision recombination reactions were conducted using linear fragments from ploxP-CCre629 as substrates (Fig. 1B). The substrates were recombined equally well when full-length Cre or both NCre and CCre were supplied (Fig. 1C). Interestingly, successful recombination was also detected in the reaction when CCre protein was used alone. While there was no related reports stating the recombination activity of CCre protein *in vitro*, and no recombination activity was detected *in vivo* in previous studies. In contrast, no recombination activity was found when only NCre was added (Fig. 1C), consistent with a previous *in vivo* study in the brain of transgenic mice [20], [21].

### Functional complementation of split-Cre in transgenic tobacco hairy roots

**Figure 2:**
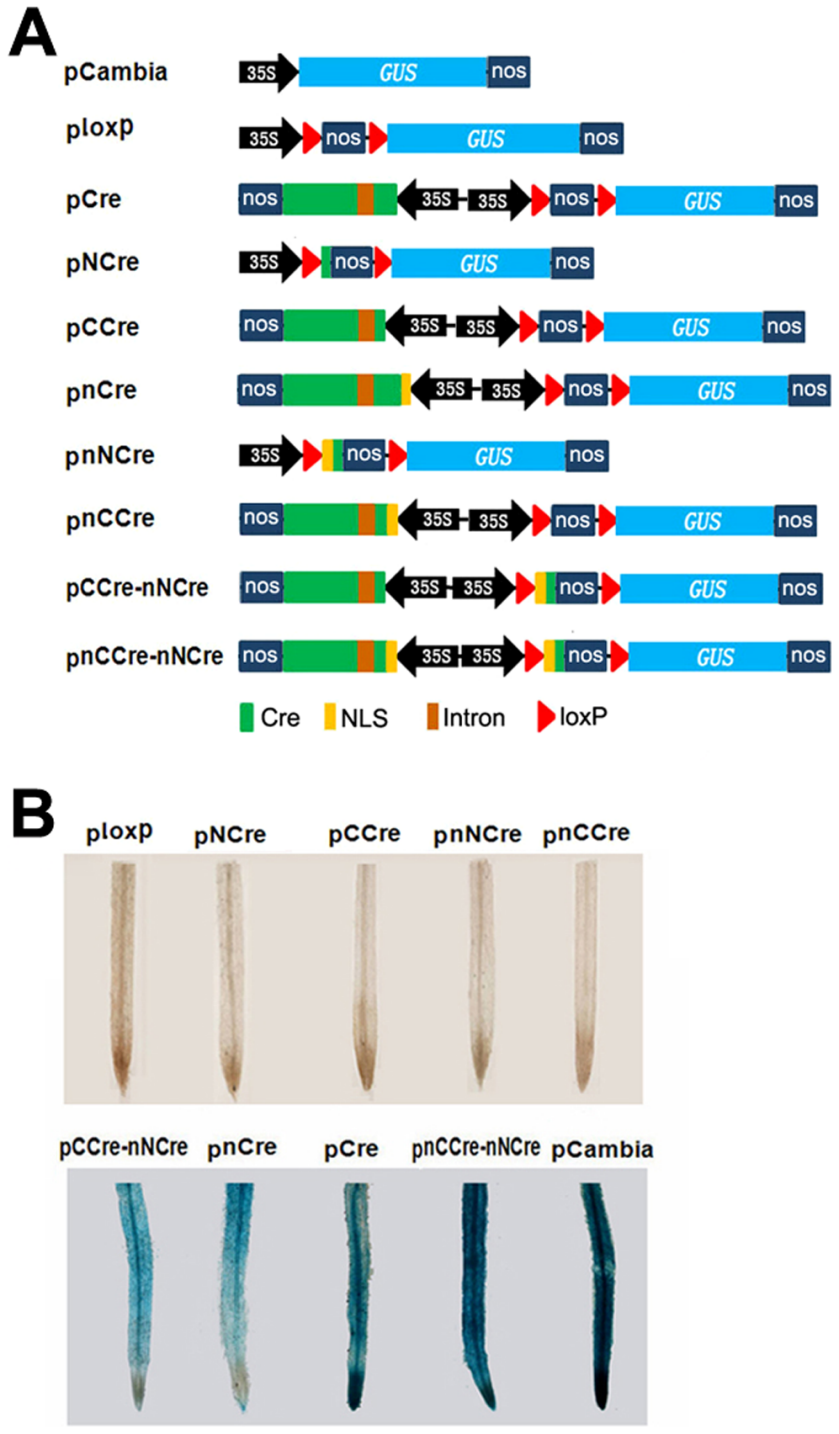
The *in vivo* recombination of split-Cre protein and the deletions determined by GUS activity. **A: Digram of plant expression vectors.** pCambia refer to vector of pCambia1305.1. **B: GUS staining of transgenic hair roots for each transformant.** “n” represents nuclear localization signal. The following are all the same.

We constructed a series of plant expression vectors for split-Cre complementation system (Fig. 2A). The plant expression vector pCAMBIA1305.1 [38], in which the *E. coli gusA* gene has been replaced by *GUSPlus*, served as an empty control. These recombinant plasmids carrying the split-Cre and full-length Cre genes were generated based on the pCAMBIA1305.1 vector. The gene cassette ploxP containing *nos* terminator sequences flanked by two 34-bp loxP sites in direct orientation, was used as a negative control. pCre and pnCre, containing full-length Cre and NLS-fused Cre driven by the CaMV 35S promoter served as positive controls. The schematic diagrams of all plant binary vectors were showed in Fig. 2A. The gene cassettes pNCre and pnNCre contained NCre and NLS-fused NCre, whereas pCCre and pnCCre contained CCre and NLS-fused CCre, respectively. The gene cassette pCCre-nNCre carried CCre and NLS-fused NCre. In the gene cassette pnCCre-nNCre, a NLS was fused into N terminus of the CCre and NCre genes, respectively.

All of the recombinant plasmids were introduced into tobacco plants by *A. rhizogenes*-mediated transformation. The hair roots of *N. tabacum* transformants with hygromycin resistance were subjected to GUS staining assay. To characterize the excision efficiency of each recombination event, we used the GUS-positive ratio to calculate the excision efficiency. Table 2 showed the total number of transgenic events analyzed for each gene cassette and the number of GUS-positive roots. As showed in Fig. 2B, no GUS activity was observed in transgenic lines harboring ploxP, pNCre, pnNCre, pCCre and pnCCre, indicating that each half (NCre and CCre) of split Cre alone, even fused with an extra NLS, did not have any recombinase activity *in vivo*. In contrast, all transgenic hair roots containing pCCre-nNCre and pnCCre-nNCre displayed blue (Fig. 2B), indicating that recombination activity of Cre is present in these transgenic plants. Transgenic lines harboring the binary vectors pCAMBIA1305.1, pCre and pnCre also showed GUS activity as expected (Fig. 2B). The results demonstrated that recombination activity of intact Cre protein could be reconstituted *in vivo* when both N- and C-terminal fragments of Cre recombinase were co-expressed, whereas no recombination activity was observed when either NCre or CCre was expressed alone.

**Table 2:** GUS positive ratio of different transgenic tobacco hair roots.

Vectors	GUS (+) No. of roots (Blue)	GUS (-) No. Of roots (White)	Total	GUS positive ratio (%)
pCAMBIA1305.1	121	0	121	100
pCA-Cre	64	66	130	49.2
pCA-nCre	77	52	129	59.7
pCA-CCre-nNCre	63	74	137	46.0
pCA-nCCre-nNCre	90	44	134	67.2
pCA-LoxP	0	49	49	0
pCA-NCre	0	53	53	0
pCA-nNCre	0	46	46	0
pCA-CCre	0	57	57	0
pCA-nCCre	0	61	61	0

**Figure 3:**
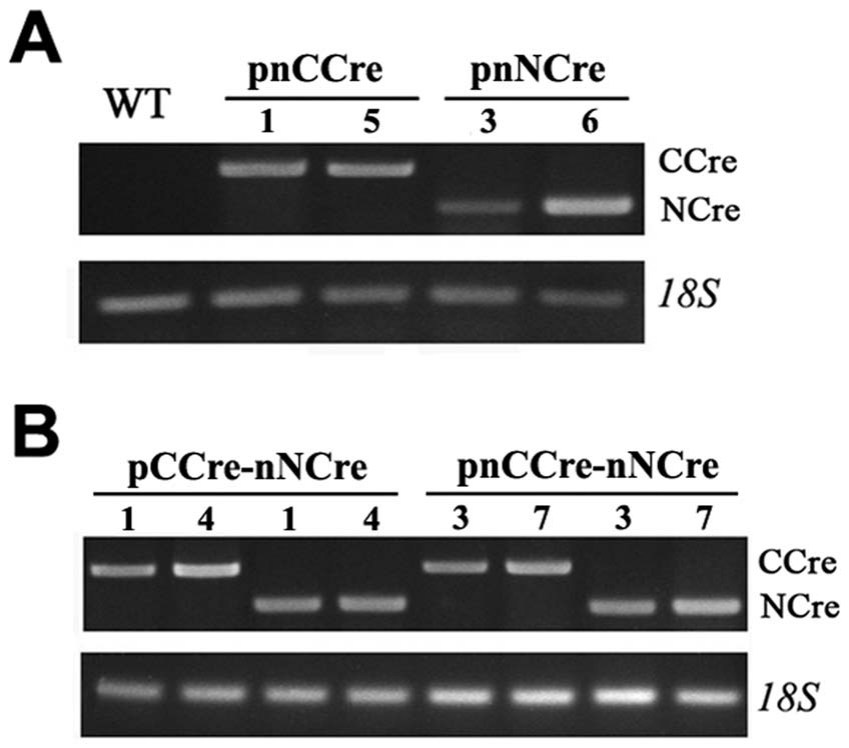
Analysis of CCre and NCre transcription in transgenic hairy roots. Semi-quantitative RT-PCR analysis of the transcription level of CCre or NCre in transformants carrying pnCCre or pnNCre (A) and pCCre-nNCre or pnCCre-nNCre (B). Tobacco 18S was used as an internal control. Total RNA was isolated from roots. Numbers represent the different lines of each recombinant.

To determine whether the split-Cre genes were indeed expressed in the hairy roots of transgenic tobacco, we used gene-specific primers to perform RT-PCR analysis. The expected DNA fragments of split-Cre recombinase were detected in these tested transformants containing pnNCre and pnCCre vectors (Fig. 3A). The *18S* rRNA complementary primers were used as an internal control. No transcripts were found in wild-type plants. Two specific PCR-amplified products for NCre and CCre were obtained in transgenic lines harboring pCCre-nNCre and pnCCre-nNCre (Fig. 3B), indicating that all the split-Cre genes transformed into transgenic tobacco plants were constitutively expressed, at least on the transcriptional level, resulting in the successful deletion of the transgene fragments flanked by two loxp sites.

### Molecular characterization of site-specific DNA excision in transgenic hairy roots

**Figure 4:**
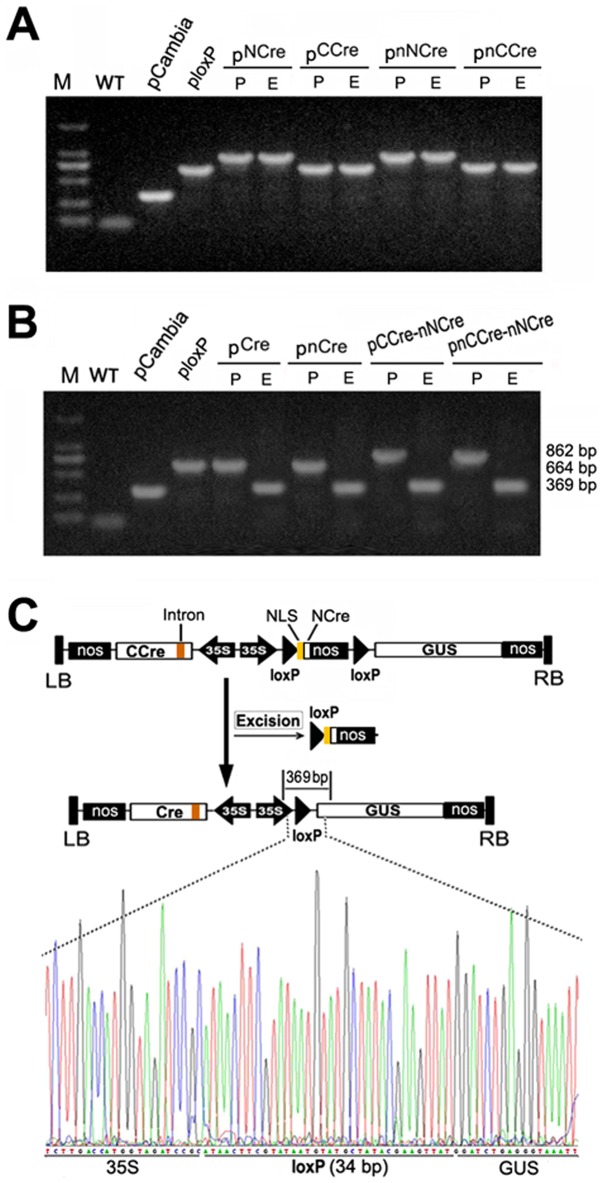
The recombinant splite-Cre excises DNA fragment between two Loxp sties *in vivo*. Vallidation of the non-excision (A) and excision (B) of DNA fragment in hairy roots. The amplified fragments of non-excision was 862 bp and 664bp for pNCre, pnNCre and pCCre, pnCCre, respectively. The amplified fragments of post-excision was 369 bp, while the pre-excision was 862 bp and 664 bp for pCCre-nNCre, pnCCre-nNCre and pCre, pnCre, respectively. M: DL2000 Marker; P, pre-excision signal; E, post-excision signal. pCambia and ploxP were used as control. **C: Schematic illustration of deletion in pCCre-nNCre and the sequencing result after deletion of DNA fragment.**

The transgene excision from hairy roots of transgenic tobacco was confirmed by PCR analysis. The genomic DNA samples extracted from different transgenic lines were used as templates for detecting the excision events. Transgenic lines carrying pCre, pnCre, pCCre-nNCre and pnCCre-nNCre vectors showed visible post-excision signals (369 bp amplification fragments) (Fig. 4B), compared to the pre-excision signals in transgenic lines pCre and pnCre (664 bp amplification fragments) and in transgenic lines pCCre-nNCre and pnCCre-nNCre (862 bp amplification fragments), respectively. No excision events were observed in transgenic lines harboring pNCre, pnNCre, pCCre, pnCCre and ploxP vectors (Fig. 4A).

Furthermore, DNA sequencing analysis revealed that the 369-bp amplification products consisted of a single loxP site and the junction T-DNA sequences located outside two loxP repeats (Fig. 4C). This result further confirmed that excision events did occur in transgenic lines pCCre-nNCre and pnCCre-nNCre, thus demonstrating that split-Cre fragments can rebuild recombination activity *in vivo* when co-expressed in plants.

### Determination of excision efficiency in the transgenic events

**Figure 5:**
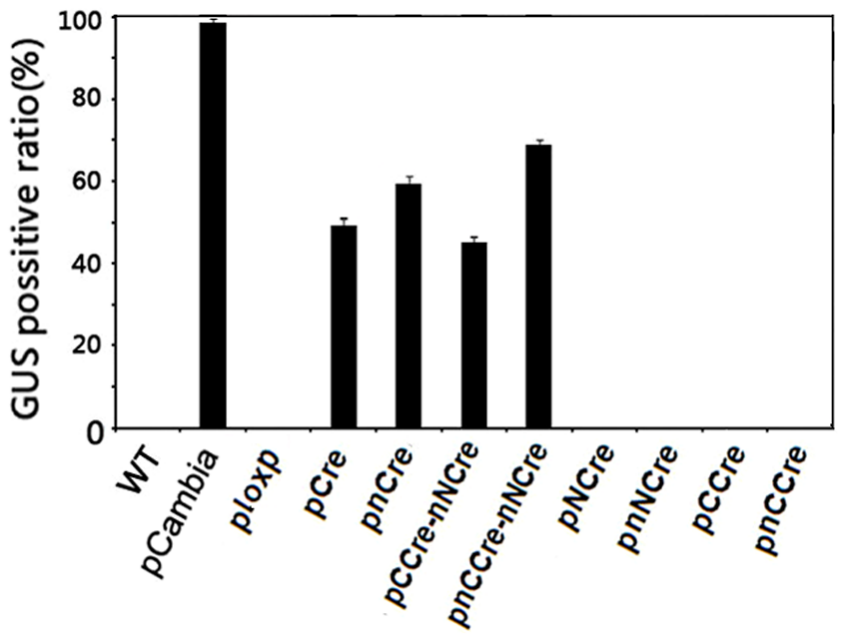
Excision ratio in the transgenic hairy roots determined by GUS staining. The ratio of GUS-positive roots was used to calculate the excision efficiencies for each transgenic line. pCAMBIA1305.1 was used as a positive control, while ploxP as a negative one. WT also used as a negative control here. All data is presented as mean of three replicates with error bars indicating ± SD.

To examine the excision efficiency of each recombinant, we analyzed the GUS positive ratio in transgenic hair roots. As shown in Fig. 5, transgenic plants containing pCAMBIA1305.1 showed strong GUS activity, whereas for transgenic lines hosting ploxP (no recombinase gene included), or pNCre, pnNCre, pCCre and pnCCre, in which each half (NCre and CCre) of split Cre was contained alone, we observed a negative GUS staining as expected. In contrast, transgenic plants hosting pCCre-nNCre (both the NCre and CCre genes expressed simultaneously) had average GUS-positive ratio of 46.0% based on a total of 137 independent transgenic events. The similar GUS-positive ratio (49.2%) was obtained in transgenic plants hosting pCre (containing the intact Cre gene). Furthermore, we found that a higher GUS positive ratio was generated in transgenic plants harboring pnCre (59.7%) and pnCCre-nNCre (67.2%) (Fig. 5), in which the nuclear localization signal (NLS) of the simian virus 40 large T antigen (SV40) was fused at the amino terminus of Cre recombinase, indicating that the NLS sequence can improve the localization of Cre recombinase to the nucleus, resulting in increasing excision efficiency by building the Cre cassette. The results demonstrated that co-expression of NCre and CCre leads to the efficient reconstitution of Cre recombinase from two inactive precursor fragments in transgenic plants.

## Discussion

The Cre/loxP recombination system has been intensively used in genetic analysis of animals and higher plants [1], [5]-[11]. One main challenge for this system is to control the expression of Cre gene in spatially and temporally desirable manners. To regulate Cre activity, in general, its expression is under the control of a cell-type specific promoter [7], [8]. However, the expression pattern of a single promoter activity is often insufficient to achieve accurate results. To overcome this limitation, split-Cre systems based on the structure of Cre recombinase have been reported previously [19], [22]. In these systems, Cre protein was generally cleaved into two complementation-competent fragments at the breakpoints in the N-terminal domain and each of these split-Cre proteins expressed alone had no enzymatic activity. But the inactive Cre moieties readily reconstituted into a functional enzyme with recombination activity when co-expressed in transgenic animals [20], [21]. In this study, the split-Cre proteins were reassembled in transgenic plants when co-expressed (Fig. 2 and 4) and the recombination efficiency was comparable to that of intact Cre recombinase (Fig. 5). However, no Cre recombination activity was detected when either NCre or CCre gene was expressed alone (Fig. 5).

The Cre/loxP system contains two elements: the Cre recombinase and two consensus sequences (loxP sites) [39]. Previous studies have reported that the C-terminal domain of the Cre recombinase harbors the active site, consisting of the conserved catalytic residues Arg173, His289, Arg292 and Trp315 [19], [40]. Furthermore, using chimeras of the Flp and Cre recombinases, Shaikh and Sadowski (2000) [41] demonstrated that the C-terminal domain of the Cre recombinase determined their mode of cleavage. In the present study, *in vitro* assays showed that NCre protein (aa 1-59) used alone was unable to catalyze DNA recombination (Fig. 1). In contrast, surprisingly, site-specific recombination events were observed when only CCre protein (the amino acid 60–343) was expressed. Previous work has shown that although a C-terminal peptide of Cre recombinase with 25 kDa still binds the loxP sites, but it is not able to catalyze the site-specific recombination [42]. Therefore, we speculated that, when lacking the small N-terminal of Cre recombinase, the presence of C-terminal domain of Cre proteins with more than 25 kDa did not affect the *in vitro* enzymatic activity of recombination.

The Cre recombinase catalyzed the site-specific recombination at two loxP sites which were located in the genomes, therefore, it has to be imported into the nucleus. Since the 38 kDa Cre protein is smaller than the ∼50 kDa upper size limit imposed by the nuclear pore on passive diffusion of macromolecules into the nucleus [43], it is hypothesized that Cre proteins enter into the nucleus by passive diffusion through the nuclear pore [44]. However, it has been reported that even small nuclear proteins of eukaryotes are more easy to gain entry to the nucleus when carrying specific nuclear localization signals (NLSs) [45]. Previous studies have demonstrated that fusing NLS sequences can effectively increase Cre recombinase activity [18], [46]. In this study, we determined the effect of NLS on the recombination activity in the split-Cre proteins. An NLS sequence from the SV40 virus was fused into the N-terminals of the NCre and CCre genes (Fig. 2). The NLS-NCre and NLS-CCre had no recombination activity in transgenic plants (Fig. 5). However, the *in vivo* recombination of the NLS-NCre and NLS-CCre proteins had higher activity compared to the wild-type Cre.

## Concluding Remarks

This study provides an alternative strategy for regulation of gene expression by site-specific recombination using the split-Cre recombinase complementation approach in plants. This system has wide application prospects in plant functional genomics and genetic engineering. In general, most of plant genes are expressed in different tissues and developmental stages. The split-Cre recombinase system allows spatial and temporal regulation of recombination through cell-specific gene genetic targeting by the simultaneous activity of two promoters in plants. In addition, a potential application of the split-Cre recombinase system is to control transgenes (i.e. selectable markers and novel trait genes) activation or removal them from transgenic plants, producing trait- or marker transgene-free transgenic crops.

## Supporting Information

Figure S1 **Prokaryotic expression and purification of split- and full-length Cre protein.** M, Protein marker. Lane 1:Induced NCre; Lane 2: Non-induced NCre; Lane 3: Induced CCre; Lane 4: Non-induced CCre; Lanes 5-6: Induced and Non-induced MBP protein as control; Lanes 7–9: Purified protein of NCre､ CCre and MBP; Lanes 10–11: Induced and Non-induced Cre; Lane 12: Purified Cre.
(TIF)
