# Supplementary figures and images for: Split-Cre Complementation Restores Combination Activity on Transgene Excision in Hair Roots of Transgenic Tobacco
Source: PLoS One. 2014 Oct 17;9(10):e110290. doi: 10.1371/journal.pone.0110290 (PMC4201524; doi:10.1371/journal.pone.0110290)

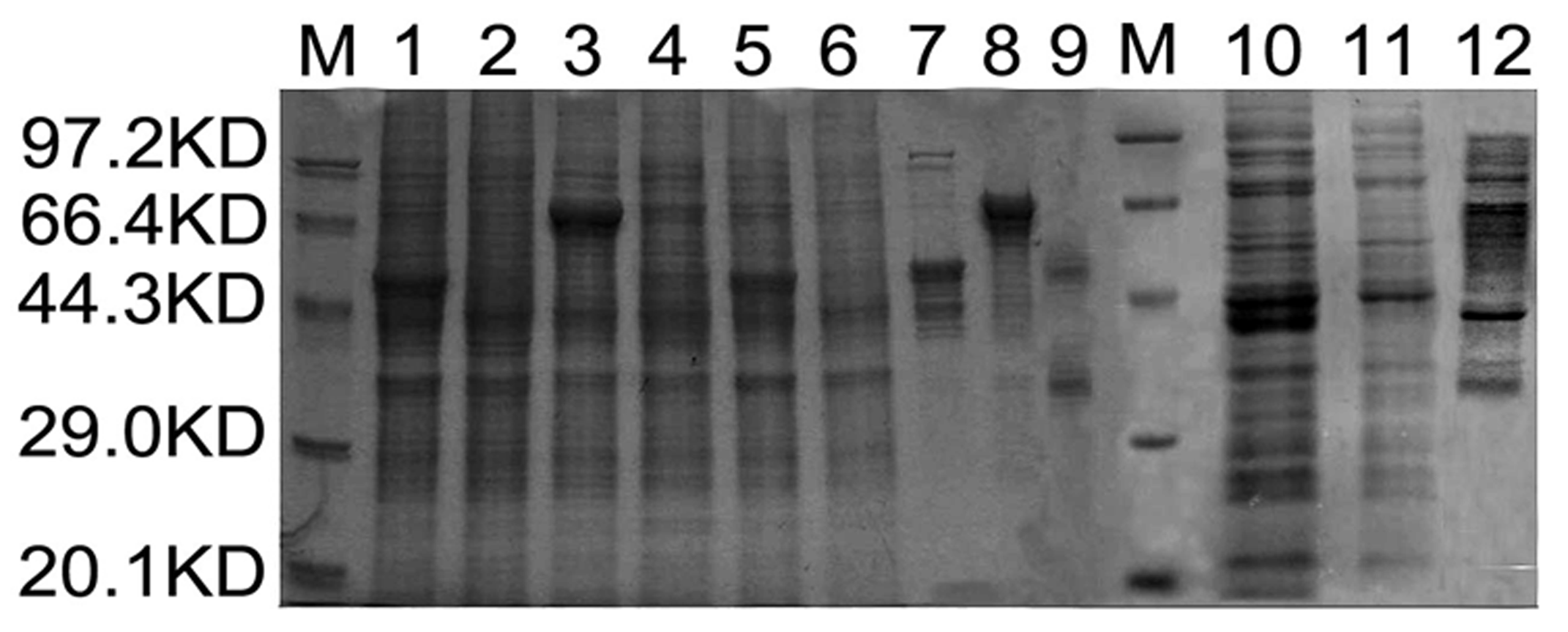

Supplement: Figure S1 — Prokaryotic expression and purification of split- and full-length Cre protein. M, Protein marker. Lane 1:Induced NCre; Lane 2: Non-induced NCre; Lane 3: Induced CCre; Lane 4: Non-induced CCre; Lanes 5-6: Induced and Non-induced MBP protein as control; Lanes 7–9: Purified protein of NCre､ CCre and MBP; Lanes 10–11: Induced and Non-induced Cre; Lane 12: Purified Cre. (TIF) [file pone.0110290.s001.tif]
